# A Novel Virus-Like Agent Originated From Genome Rearrangement of Porcine Circovirus Type 2 (PCV2) Enhances PCV2 Replication and Regulates Intracellular Redox Status *In Vitro*

**DOI:** 10.3389/fcimb.2022.855920

**Published:** 2022-04-13

**Authors:** Huicheng Feng, Jinping Fu, Bo Zhang, Tao Xue, Chuanmin Liu

**Affiliations:** ^1^ School of Pharmacy, Linyi University, Linyi, Shandong, China; ^2^ Institute of Veterinary Medicine, Jiangsu Academy of Agricultural Sciences, Nanjing, China; ^3^ Key Laboratory of Veterinary Biological Engineering and Technology, Ministry of Agriculture, Nanjing, China; ^4^ Key Laboratory of Veterinary Diagnosis, Jiangsu Academy of Agricultural Sciences, Nanjing, China; ^5^ School of Life Sciences, Jiangsu University, Zhenjiang, China; ^6^ College of Veterinary Medicine, Nanjing Agricultural University, Nanjing, China

**Keywords:** virus-like agent, genome rearrangement, porcine circovirus type 2, viral replication, redox status

## Abstract

Genome rearrangement occurs to porcine circovirus type 2 (PCV2) during *in vitro* and *in vivo* infections, and a number of rearranged PCV2 genomes have been isolated and characterized. This study was conducted to investigate the role of the rearranged PCV2 (rPCV2) in PCV2 replication and the biological effect of rPCV2 in host cells. Two whole rPCV2 genome sequences (358 nt and 1125 nt in length) were synthesized and recombinant plasmids pBSK(+)-rPCV2 (pBSK(+)-1125 and pBSK(+)-358) were constructed. A novel virus-like agent (rPCV2-1125) was rescued by *in vitro* transfection of porcine kidney cell line (PK-15) and porcine alveolar macrophage 3D4/21 cells. The data indicate that rPCV2-1125 significantly enhanced PCV2 replication *in vitro*. Furthermore, rPCV2-1125 led to oxidative stress in host cells, as indicated by decreased intracellular glutathione (GSH) and total superoxide dismutase (SOD) activities, as well as increased malondialdehyde (MDA) levels. These results provide new insights into genome rearrangement of PCV2 and will contribute to future studies of PCV2 replication and associated mechanisms.

## Introduction

Porcine circovirus (PCV), belonging to the genus *Circovirus* in the family Circoviridae, was first recognized as a contaminant of the continuous porcine kidney cell line (PK-15) in 1974 in Germany (Ellis, 2014). It is a group of small, nonenveloped viruses with a circular, single-stranded DNA genome (Tischer et al., 1982). Four genotypes of PCV are known to us, including non-pathogenic PCV type 1 (PCV1) (Tischer et al., 1974), pathogenic PCV type 2 (PCV2) (Segalés et al., 2008), recently identified PCV type 3 (PCV3) (Palinski et al., 2016; Phan et al., 2016) and PCV type 4 (PCV4) (Zhang et al., 2020). PCV2 is the causative agent of PCV associated disease (PCVAD), which has emerged globally and caused great economic losses for the global swine industry and swine-producing countries (Chae, 2005; Gillespie et al., 2009; Zhai et al., 2014; Saporiti et al., 2020; Wang et al., 2020; Zheng et al., 2020).

In virology, defective interfering particles (DIPs), also known as defective interfering viruses, are spontaneously generated virus mutants in which a critical portion of the genome is lost due to defective replication (Marriott and Dimmock, 2010). The existence of DIPs has been known for decades, and they can occur in nearly every class of both DNA and RNA viruses (Stauffer Thompson et al., 2009; Dimmock et al., 2012; Saira et al., 2013; Sun et al., 2015; Petterson et al., 2016). In recent years, a variety of rearranged genomic PCV2 subviral molecules have been demonstrated *in vivo* and *in vitro*, and are regarded as PCV2 DIPs (Wen et al., 2012a; Wen et al., 2012b; Wen et al., 2012c; Wen et al., 2012d; Huang et al., 2012; Wen et al., 2012e). Sequencing of the PCV2 DIPs showed that their lengths ranged from 436 nt to 896 nt, for those obtained from sera samples of PCV2-infected pigs, and from 358 nt to 1125 nt, for those obtained from PCV2-infected PK-15 cells (Wen et al., 2013). However, Wen et al. (2013) pointed out that PCV2 DIPs were difficult to identify using an electron microscope because of the difficulty in purifying rearranged PCV2 (rPCV2) free of the wildtype PCV2 (Wen et al., 2013). Besides, the effect of rPCV2 on replication of wildtype PCV2 and the biological activity of rPCV2 in host cells remain unclear.

In this study, the whole genome sequences of two rPCV2 DIPs (358 nt and 1125 nt in length) were synthesized and rPCV2 was rescued *in vitro*. For the first time, PCV2 DIPs were successfully detected using an electron microscope. In addition, the effects of rPCV2 on PVC2 replication and intracellular redox status were also investigated.

## Materials and Methods

### Cell Lines, Virus and Sequences of rPCV2

PK-15 and 3D4/21cells, free of PCV, were purchased from the China Institute of Veterinary Drug Control (Beijing, China) and grown at 37°C in an atmosphere of 5% CO2 in Dulbecco’s modified Eagle’s medium (DMEM) (Sigma, St. Louis, MO, USA) supplemented with 10% fetal bovine serum (FBS) (Gibco, Waltham, MA, USA) and 1% penicillin-streptomycin antibiotics (Sangon, China). The PCV2-Haian strain (GenBank accession number FJ712216.1) maintained by the Institute of Veterinary Medicine, Jiangsu Academy Agricultural Sciences, was propagated in PK-15 cells, harvested after 72 h incubation, and stored at −70°C until use. The genome sequences of PCV2-Haian strain, rPCV2-1125, and rPCV2-358 were obtained from GenBank (accession numbers FJ712216.1, JX984589, and JX984585, respectively).

### Construction of Recombinant Plasmids Containing the rPCV2 Sequences

The whole genome sequences of rPCV2-1125 and rPCV2-358 were synthesized by Shanghai Majorbio Biomedicine Technology Co., Ltd., China. Recombinant pBSK(+)-1125 and pBSK(+)-358 plasmids were generated by cloning two tandem copies of the complete rPCV2-1125 or rPCV2-358 sequence into a pBluescriptII SK(+) vector, as described previously (Fenaux et al., 2002). The recombinant plasmids were transformed into *E. coli* DH5α and positive colonies were screened by ampicillin. Plasmid DNA was extracted using an Endo-free Plasmid Mini Kit II (Omega Bio-Tek, Norcross, GA, USA) according to the manufacturer’s instructions, and confirmed by DNA sequencing followed with analysis using DNAMAN 9.0 software (Wang et al., 2021).

### Cell Transfection

PK-15 and 3D4/21 cells were seeded into 24-well tissue culture plates and grown to approximately 60%-80% confluency. After washing with OptiMEM medium (Gibco, Waltham, MA, USA), cells were transfected with the recombinant pBSK(+)-1125 or pBSK(+)-358 plasmid using Lipofectamine^®^ 2000 (Invitrogen, Carlsbad, CA, USA) according to the manufacturer’s protocol. The cells were then replaced with complete medium (DMEM with 10% FBS) 6 h post transfection.

### Morphological Observations of the Rescued Viruses

Cell samples with rescued rPCV2 were harvested, frozen and thawed three times, and ultracentrifuged in CsCl for 48 h at 270000 g. The product from the CsCl gradient centrifugation were negatively stained with 2% phosphotungstic acid for 60 s. Morphological characteristics of the virus were observed under a transmission electron microscope (Philips, Amsterdam, Netherlands) (Wen et al., 2012e).

### Indirect Fluorescence Assay (IFA)

The PCV2-based IFA was performed as described previously (Liu et al., 2011). In brief, PK-15 and 3D4/21 cells were fixed with cold methanol at 4°C for 10~15 min. After washing with PBS, the fixed cells were incubated with optimally diluted porcine anti-PCV2 antibody (VMRD, Pullman, WA, USA) at 37°C for 1 h, washed with PBS again and incubated with FITC-conjugated goat anti-pig antibody (Abcam, UK) at 37°C for 45 min. Then, the stained cells were washed with PBS and examined under a fluorescence microscope (Olympus, Shinjuku, Tokyo, Japan).

### Virus Titers

Samples collected from the cell culture plates were frozen and thawed three times, and then serially ten-fold diluted in DMEM (1:10 to 1:10^8^). The cells free of PCV were seeded in 96-well plates, grown until reaching 40%-50% confluency, and then inoculated with the diluted samples, with eight wells for each dilution. After 48 h incubation, PCV2-positive wells in each 96-well plate were determined by IFA as described previously (Liu et al., 2011), and virus titers were calculated by the Reed-Muench method (Reed and Muench, 1938). Each experiment was performed in triplicate.

### DNA Extraction and TaqMan-Based Real-Time PCR

DNA was extracted using a Column Viral DNAout Kit (TIANDZ, Beijing, China) according to the manufacturer’s instructions. Total DNA was stored at -70°C until use. To analyze viral DNA, the primers and TaqMan probe specific to wildtype PCV2 (rPCV2 would not be detected) were designed as follows:

forward primer 5’- AGTGAGCGGGAAAATGCAGA -3’,
reverse primer 5’- TATGTGGTTTCCGGGTCTGC -3’,
TaqMan probe 5’-[6-FAM] ATTGTGGGGCCACCTGGGTG [TAMRA]-3’.

A recombinant pGEM-T easy vector (TaKaRa, China) containing PCV2 genome insert was constructed, as described previously (Xue et al., 2019). The qPCR standard curve was generated by analysis of ten fold serial dilutions ranging from 10^2^ to 10^7^ copies. Each reaction was run in triplicate. TaqMan-based real-time PCR was performed on 7500 Real-Time PCR Systems (Applied Biosystems, Bedford, MA, USA) by using the following thermal cycles: 95°C for 30 s, 40 cycles at 95°C for 5 s, and 60°C for 34 s.

### Detection of GSH, SOD and MDA

GSH, SOD and MDA were determined by spectrophotometry using commercial kits obtained from Nanjing Jiancheng Bioengineering Institute, China. The catalog numbers for GSH, SOD and MDA assay kits are A006-2, A001-3 and A003-1, respectively. Briefly, cell samples were collected, homogenized with ice cold saline and centrifuged at 12,000 × g at 4°C for 20 min. The resulting supernatants were used for biochemical assessments according to the manufacturer’s protocols. At the same time, total cellular protein was determined by Coomassie blue staining with total protein quantitative assay kit (catalog number A-045-2) (Nanjing Jiancheng Bioengineering Institute, Nanjing, China) according to the manufacturer’s instructions. Each experiment was performed in triplicate.

### Statistical Analysis

Tests of significance were performed using Duncan’s multiple-range test after one-way ANOVA with the SPSS 23.0 statistics software (Lim et al., 2021). Data are presented as means ± SD. A value of *P* < 0.05 was considered statistically significant.

## Results

### Identification of Recombinant Plasmids

**Figure 1:**
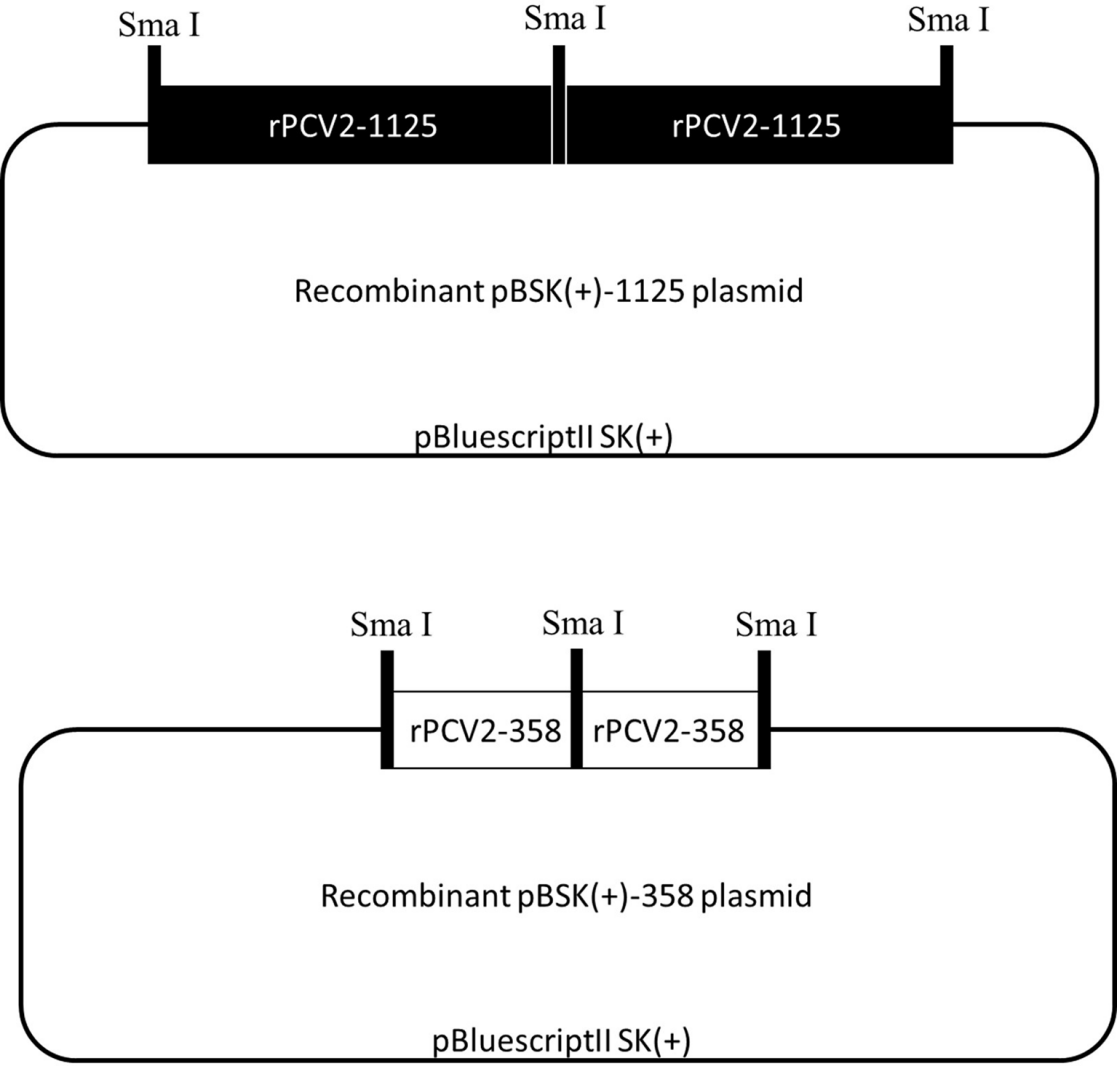
Schematic diagrams of recombinant plasmids. Recombinant pBSK(+)-1125 and pBSK(+)-358 plasmids were constructed by inserting two tandem copies of the complete rPCV2-1125 or rPCV2-358 genome into the pBluescriptII SK(+) vector.

Schematic diagrams of the recombinant pBSK(+)-1125 and pBSK(+)-358 plasmids were displayed in **Figure 1**, where two tandem copies of rPCV2-1125 or rPCV2-358 genome were cloned into the pBSK(+) vector (2977 bp in length). The dimerized tandem DNA clone is more efficient in transfecting cells than the clone containing one single copy of the viral genome (Fenaux et al., 2002). The recombinant pBSK(+)-1125 and pBSK(+)-358 plasmids were both confirmed by DNA sequencing (data not shown).

### Morphological Observations

**Figure 2:**
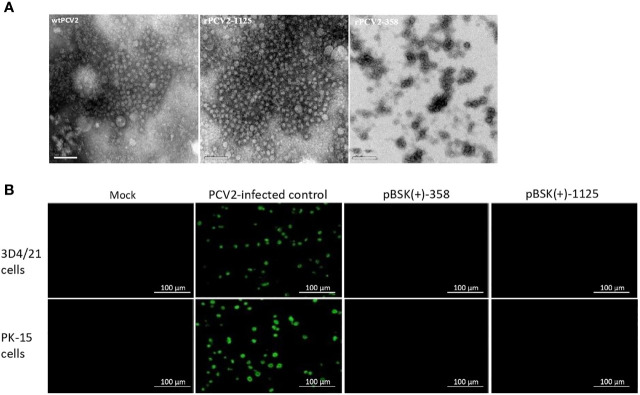
Detection of rPCV2 under microscope. **(A)** Rescued viruses examined by electron microscopy (bar=100 nm). PCV2 virus-like particles were observed using negative staining in rPCV2-1125 samples. **(B)** PCV2-based IFA only specifically detected wildtype PCV2 but not rPCV2. No fluorescence signal was detected in cells transfected with either pBSK(+)-1125 or pBSK(+)-358.

Rescued rPCV2-1125 particles with a uniform shape (approximate 20 nm in diameter), which is similar to the size of wildtype PCV2 (Mo et al., 2019), were observed under an electron microscope (**Figure 2A**). In contrast, no virus-like particle was detected from the cells transfected with pBSK(+)-358 (**Figure 2A**).

### IFA Detection of rPCV2

PCV2-based IFA only specifically detected wildtype PCV2 (**Figure 2B**). No fluorescence signal was detected in cells transfected with either pBSK(+)-1125 or pBSK(+)-358 (**Figure 2B**).

### Effects of rPCV2 on PCV2 Replication

**Figure 3:**
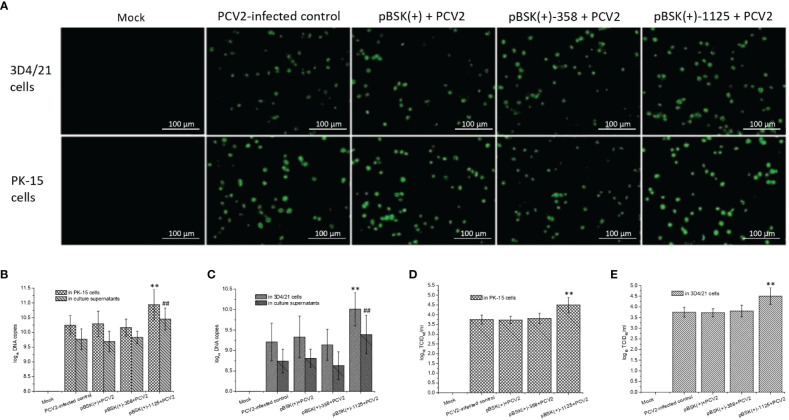
Effects of rPCV2 on PCV2 replication *in vitro*. Cells were seeded into 24-well tissue culture plates and grown to 60%-80% confluency, and then transfected with the recombinant pBSK(+)-1125 or pBSK(+)-358 plasmids. After 6 h, the cells were infected with PCV2 (multiplicity of infection (MOI) of 1.0) for 48 h. By IFA, PCV2-specific fluorescence was examined under a fluorescence microscope **(A)** (200× magnification). Samples from the cell extracts and the supernatants were harvested separately. PCV2 DNA copies **(B, C)** and virus tiers **(D, E)** were determined. Noninfected cells were considered as the mock control while the cells infected with PCV2 alone were used as PCV2-infected control. Data are presented as means ± SD from three independent experiments. In each cell line or in culture supernatant, ^**^ *P < *0.01 and ^##^ *P < *0.01 for transfected cells or culture supernatant vs the PCV2-infected control. Each experiment was performed in triplicate.

PCV2-based IFA (**Figure 3A**), to some degree, suggest that transfection with pBSK(+)-1125 likely led to increased PCV2 infection, which was further supported by the measurement of viral DNA copies and virus titers. PCV2 DNA copy numbers from PCV2-infected cells transfected with pBSK(+)-1125 or from the culture supernatants of these cells were significantly higher than the DNA copy numbers from the PCV2-infected control (*P* < 0.01) (**Figures 3B, C**). Similarly, virus titers from PCV2-infected cells transfected with pBSK(+)-1125 were also significantly increased compared with virus titers from the PCV2-infected control (*P* < 0.01) (**Figures 3D, E**). Moreover, transfection with pBSK(+)-358 or pBSK(+) had very little effect on PCV2 replication. Data obtained from the PK-15 and 3D4/21 cells were consistent, indicating that rPCV2-1125 significantly enhanced PCV2 replication *in vitro*.

### Effects of rPCV2 on the Intracellular Redox Status

**Figure 4:**
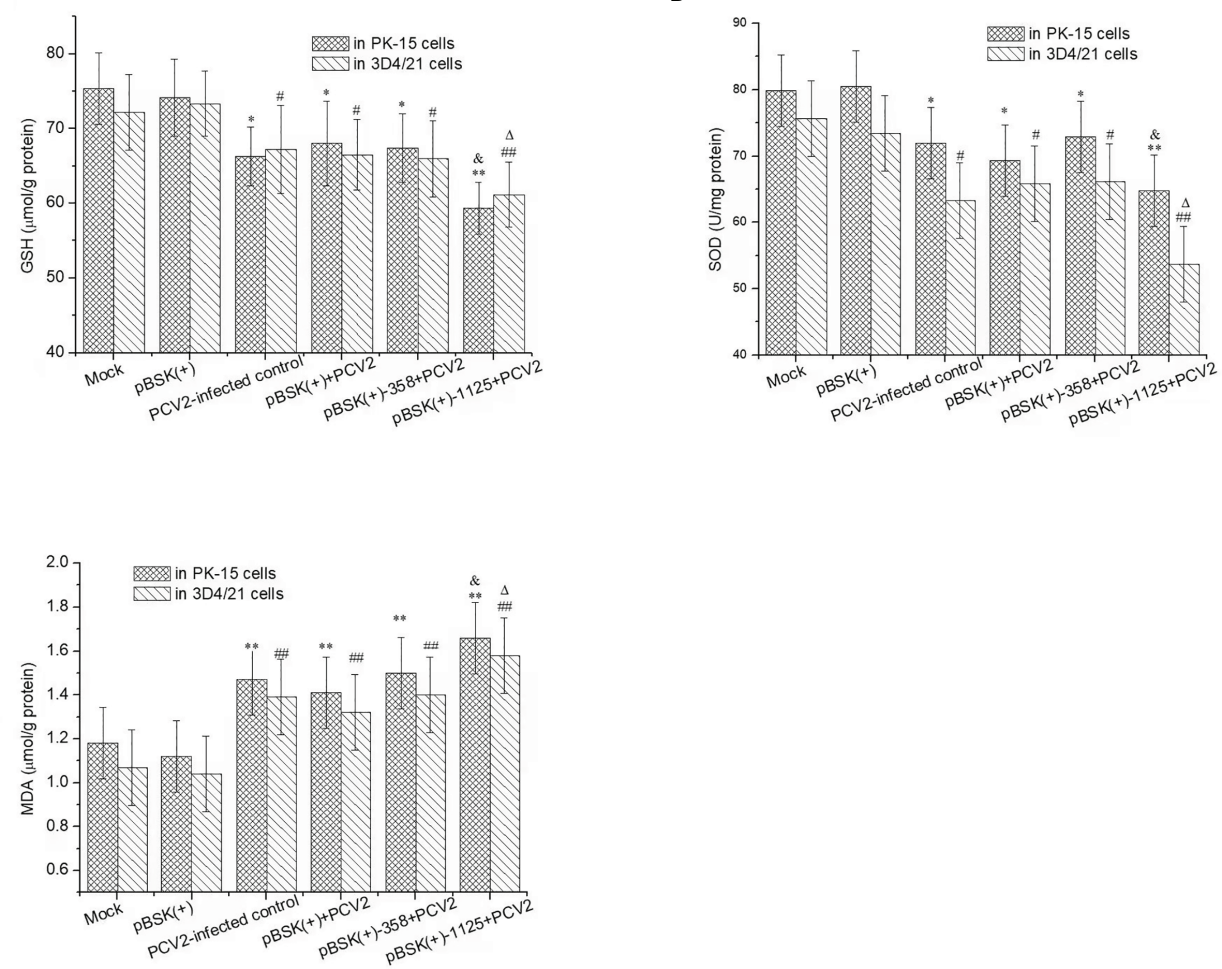
The intracellular redox status regulated by rPCV2. Cells were seeded into 24-well tissue culture plates and grown to 60%-80% confluency, and then transfected with the recombinant pBSK(+)-1125 or pBSK(+)-358 plasmids. 6 h post transfection, the cells were infected with PCV2 (1 MOI) for 48 h Cell culture supernatants were removed and the cell samples were washed with PBS, and then trypsinized and harvested separately. GSH **(A)**, SOD **(B)** and MDA **(C)** were detected by the commercial kits. Data are presented as means ± SD from three independent experiments. In the same cell line, ^*^ *P < *0.05, ^**^ *P < *0.01, ^#^ *P <*0.05, and ^##^ *P < *0.01 for transfected cells vs mock cells; ^&^ *P < *0.05 and ^△^ *P < *0.05 for transfected cells vs the PCV2-infected control. Each experiment was performed in triplicate.

PCV2 infection resulted in a marked decrease in GSH and SOD activity and an increase in MDA levels in PK-15 and 3D4/21 cells (*P* < 0.05) (**Figure 4**). Moreover, GSH and SOD activity in PCV2-infected cells transfected with pBSK(+)-1125 were significantly lower than those in the mock control cells or PCV2-infected control cells (*P* < 0.05 or *P* < 0.01). Nevertheless, MDA levels in PCV2-infected cells transfected with pBSK(+)-1125 were significantly higher than those in mock cells or in PCV2-infected control cells (*P* < 0.05 or *P* < 0.01). However, GSH and SOD activity and MDA level in the PCV2-infected cells transfected with pBSK(+) or pBSK(+)-358 were similar to those in the PCV2-infected control cells (*P* > 0.05). Additionally, transfection with pBSK(+) alone almost did not alter intracellular GSH, SOD or MDA in relation to those in mock control cells.

## Discussion

PCV2 is the causative agent of PCVAD which is a multifactorial disease linked to both non-infectious and infectious factors (Opriessnig and Halbur, 2012; Sun et al., 2016). Postweaning multisystemic wasting syndrome (PMWS), the first recognized PCVAD in Canada in 1991 (Clark, 1997), is one of the most important PCVADs affecting the pig industry. It is difficult to reproduce PMWS by experimental infection with PCV2 alone, because the development of full-blown PMWS induced clinical symptoms and pathological lesions requires other co-infection factors (Chang et al., 2005), including some unknown pathogens. Our research group previously reported that a porcine circovirus-like agent P1, considered as a PCV2 recombinant with other organisms rather than intragenomic rearrangement, caused PMWS symptoms in pigs. Interestingly, the disease caused by P1 was different from PCV2-induced PMWS (Wen et al., 2012e). We have described many rearranged genomes of PCV2 (Wen et al., 2013; Wen and He, 2021), but, to date, only the rPCV2 sequences have been analyzed and no further studies on rPCV2 have been performed.

The PCV2 genome consists of 1767 or 1768 nucleotides with 11 putative open reading frames (ORFs), only five of which have been confirmed to encode proteins (Lv et al., 2015). ORF1 encodes a replicase (Rep), which is considered as the essential protein for viral replication (Cheung, 2003). ORF2 encodes a capsid protein (Cap), which is the only structural protein of PCV2 and contains a nuclear location signal in the N-terminus (Nawagitgul et al., 2000). ORF3 encodes a non-structural protein that has been reported to associate with viral replication and pathogenesis (Karuppannan et al., 2009). ORF4 encodes a protein that plays a role in suppressing the PCV2-induced apoptosis, but is not essential for PCV2 replication (He et al., 2013). ORF5 encodes a novel protein that is not essential for viral replication and likely plays an important role in persistent PCV2 infection by regulating the NF-κB signaling pathway (Lv et al., 2015). There is a deletion of more than 600 nucleotides in rPCV2-1125 genome. Sequence alignment suggested that the rPCV2-1125 genome contains a truncated ORF1, whole ORF2, parts of ORF4 and ORF9, whereas the rPCV2-358 genome contains only a truncated ORF1 and part of ORF5. The truncated ORF1 in rPCV2-1125 contains replication origin and replication motifs I, II and III (Mankertz et al., 2004), however, it lacks middle and N-terminal parts that are essential for replication functions, including oligomerization and ATPase domains (Tarasova et al., 2021). Moreover, the truncated ORF1 in rPCV2-358 retains none of the motifs or domains described above. The whole ORF2 in rPCV2-1125 contains few point mutations leading to 1% difference in the alignment with wild type PCV2 capsid protein sequence. The antigenic epitopes recognized by polyclonal antibodies may be partially located in the 1% differential region, which to some degree explains the IFA experiment failed for rPCV2-1125 detection (Larochelle et al., 2002; Ge et al., 2013). In the current study, we constructed recombinant pBSK(+)-1125 and pBSK(+)-358 plasmids and successfully rescued rPCV2-1125 by *in vitro* transfection. Rescued rPCV2-1125 particles were observed by electron microscopy, but no virus-like particles were detected from the cells transfected with pBSK(+)-358. These findings confirmed that ORF2 was essential for formation of porcine circovirus-like particles. However, propagation of rPCV2-1125 in serial passage cells was limited because rPCV2-1125 is a DIP with a truncated ORF1 which affected viral replication. Previous reports also revealed that DIPs could still penetrate host cells, but required another fully functional virus particle (the ‘helper’ virus) to co-infect the cells, in order to compensate the lost factors (Makino et al., 1988; Palmer et al., 2011).

Co-infection with other infectious agents, such as porcine parvovirus (PPV) (Allan et al., 1999), porcine reproductive and respiratory syndrome virus (PRRSV) (Allan et al., 2000) and Mycoplasma hyopneumoniae (Opriessnig et al., 2004), or stimulation with non-infectious agents, such as lipopolysaccharide (Chang et al., 2006), concanavalin A (Lefebvre et al., 2008) and the interferons IFN-α/IFN-γ (Ramamoorthy et al., 2009), remarkably enhanced PCV2 replication *in vitro* or *in vivo*. Interestingly, rPCV2-1125 was proved to effectively enhance PCV2 replication in the present study, as indicated by the remarkable increase in PCV2 DNA copies and virus titers in PK-15 and 3D4/21 cells. Although the mechanisms associated with this phenomenon were unclear, we accidentally found that intracellular redox status was regulated by rPCV2-1125, because *in vitro* transfection with pBSK(+)-1125 significantly decreased the GSH and SOD activity and increased the MDA level in PCV2 infected PK-15 and 3D4/21 cells. A possible relationship between PCV2 infection and host oxidative stress was suggested previously (Chen et al., 2012a). Indeed, PCV2 infection induces oxidative stress in host cells while oxidants, such as H_2_O_2_, mediates oxidation which contributes to an increase in PCV2 replication (Chen et al., 2012b; Chen et al., 2013). Data from this study indicates that enhanced PCV2 replication might be partly due to oxidative stress caused by rPCV2-1125, which provides a potential mechanism for the regulation of PCV2 replication induced by rPCV2-1125. However, genes, proteins and signaling pathways involved in the regulation process remain to be discovered. Moreover, *in vivo* studies are needed to investigate the effects of rPCV2-1125 on PCV2 infection in pigs.

## Conclusion

A novel virus-like agent named rPCV2-1125, originated from the intragenomic rearrangement of PCV2, was rescued *in vitro* for the first time. We also demonstrated that rPCV2-1125 notably enhanced PCV2 replication *in vitro*. This phenomenon was partly due to changes in the intracellular redox status of infected cells, because we found that oxidative stress caused by rPCV2-1125 was closely linked to PCV2 replication in the host cells.

## Data Availability Statement

The original contributions presented in the study are included in the article/supplementary material. Further inquiries can be directed to the corresponding authors.

## Author Contributions

CL and TX directed the research and reviewed the data and manuscript. CL wrote the manuscript. HF and JF conducted the research and compiled the data. BZ was involved in data analysis and participated in drafting the manuscript. All authors have read and approved the manuscript.

## Funding

This work was supported by grants from Shandong Provincial Natural Science Foundation, China (ZR2020MC019, ZR2021LZY030), the National Natural Science Foundation of China (31502099). Open access publication fees will be supported by the grant from Shandong Provincial Natural Science Foundation, China (ZR2020MC019).

## Conflict of Interest

The authors declare that the research was conducted in the absence of any commercial or financial relationships that could be construed as a potential conflict of interest.

## Publisher’s Note

All claims expressed in this article are solely those of the authors and do not necessarily represent those of their affiliated organizations, or those of the publisher, the editors and the reviewers. Any product that may be evaluated in this article, or claim that may be made by its manufacturer, is not guaranteed or endorsed by the publisher.
